# Tissue transglutaminase treatment leads to concentration-dependent changes in dendritic cell phenotype - implications for the role of transglutaminase in coeliac disease

**DOI:** 10.1186/1471-2172-13-20

**Published:** 2012-04-16

**Authors:** William J Dalleywater, David YS Chau, Amir M Ghaemmaghami

**Affiliations:** 1 Allergy and Tissue Modelling Research Group, School of Molecular Medical Sciences, Queen's Medical Centre, The University of Nottingham, A Floor, West Block, Nottingham, NG7 2UH, UK

**Keywords:** Coeliac disease, Dendritic cells, Immune response, Gliadin, Tissue engineering, Transglutaminase

## Abstract

Dendritic cells (DCs) are part of the innate immune system with a key role in initiating and modulating T cell mediated immune responses. Coeliac disease is caused by inappropriate activation of such a response leading to small intestinal inflammation when gluten is ingested. Tissue transglutaminase, an extracellular matrix (ECM) protein, has an established role in coeliac disease; however, little work to date has examined its impact on DCs. The aim of this study was to investigate the effect of small intestinal ECM proteins, fibronectin (FN) and tissue transglutaminase 2 (TG-2), on human DCs by including these proteins in DC cultures.

The study used flow cytometry and scanning electron microscopy to determine the effect of FN and TG-2 on phenotype, endocytic ability and and morphology of DCs. Furthermore, DCs treated with FN and TG-2 were cultured with T cells and subsequent T cell proliferation and cytokine profile was determined.

The data indicate that transglutaminase affected DCs in a concentration-dependent manner. High concentrations were associated with a more mature phenotype and increased ability to stimulate T cells, while lower concentrations led to maintenance of an immature phenotype.

These data provide support for an additional role for transglutaminase in coeliac disease and demonstrate the potential of *in vitro *modelling of coeliac disease pathogenesis.

## Background

Dendritic cells (DCs) are part of the innate immune system with a vital role in modulating adaptive immune responses [1,2]. DCs have a life cycle consisting of two distinct phases [2]. In the immature state, they act as sentinels and are particularly concentrated in areas of microbial exposure, where they take up and process antigens for presentation by MHC molecules [3]. Once activated, by ligation of pattern-recognition receptors, they begin to mature and migrate to local lymph nodes [2]. In lymph nodes, they present antigens to specific T-helper (Th) cells that, together with other factors such as cytokine microenvironment, determine the differentiation of T cells to one of several specialised subsets, such as Th1, Th2, Th17 or Treg. [2,4]. DCs are particularly important for the induction of naïve Th responses owing to their abundant surface expression of co-stimulatory molecules such as CD80 and CD86 [3-5]. DCs are also thought to play an important role in inducing and controlling tolerance in the periphery [6]. T cells that interact with immature DCs, which have low surface expression of co-stimulatory molecules [2], are likely to undergo apoptosis, become anergic (immunologically unresponsive) or differentiate to a regulatory phenotype [6].

DCs have an important role in modulating T cell mediated immune responses [2], and therefore factors affecting their function have significance for all adaptive immune responses. A recent study [1] found that extracellular matrix (ECM) components, particularly laminin and fibronectin (FN), have an impact on DC phenotype and function. DCs interact with ECM via integrins and it is suggested that through this signalling pathway, laminin and FN maintain DCs in an immature phenotype, with high surface expression of endocytic receptors and low expression of molecules needed for T cell signalling [1,7]. Another ECM protein with a putative role in determining DC phenotype is tissue transglutaminase 2 (TG-2) [8]. TG-2 is a member of the transglutaminase enzyme family, which comprises several important enzymes involved in protein cross-linking [9]. TG-2 is already known to have a role in DC function [8,10], although the full details of its role are yet to be determined. DCs have been found to increase the expression of TG-2 as their life cycle progresses, with particularly high levels during the final stages of maturation [10]. TG-2 may also play a part in DC maturation in response to LPS stimulation; recent research found increased levels of TG-2 subsequent to encountering LPS [8]. This research also indicated that DCs in mice lacking TG-2 may be unable to fully mature (following LPS stimulation), and therefore have reduced capacity to stimulate CD4+ T cell, in particular Th1, responses [8]. These findings provide interesting insights into the potential role of TG-2 in DC function and maturation, but give considerable scope for further investigation into the nature of its part in determining signals delivered by DCs to T cells and the resulting effect on T cell mediated responses.

TG-2 is also involved in the pathogenesis of coeliac disease [11-13], with well-established roles both in increasing the immunogenicity of gluten antigens and also as an autoantigen [11], against which autoantibodies are directed. Coeliac disease is an immunological condition, provoked by ingestion of gluten, in which inflammation of the small intestine (in genetically predisposed individuals) is mediated by T cells specific for gluten-derived antigens [13]. Interestingly, previous studies have indicated that T cells specific for these antigens can be found in the peripheral blood of both healthy individuals as well as coeliac disease patients [14,15]. DCs serve as the major antigen presenting cells to T cells [2], stimulating protective and, in the case of coeliac disease, pathological adaptive immune responses. TG-2 is expressed in the small intestine, with raised levels in coeliac disease [12], and it is therefore possible that TG-2 interacts with DCs to contribute to the pathogenesis of coeliac disease, for example, by increasing the ability of DCs to stimulate T cell-mediated responses.

The aim of this study was to characterise the effect of TG-2 on DCs. By including TG-2 in monocyte derived DC cultures at early stages of their differentiation, the study sought to investigate what effect it might have on DC phenotype, morphology and function. The study also aimed to investigate the role of TG-2 in coeliac disease by assessing whether TG-2 affected the ability of DCs to stimulate naïve T cells in response to gliadin, the major antigen found in gluten.

## Methods

### Ethics statement

Peripheral blood mononuclear cells were separated from buffy coat preparations obtained from National Blood Service (Sheffield, UK) following institutional ethics guidelines.

### Generating DCs

Monocyte isolation and generation of DCs was carried out as described previously [1]. Briefly, heparinised whole blood was layered on Histopaque (Sigma, Irvine, UK) and centrifuged in order to fractionate blood for collection of PBMCs. Monocytes were isolated from PBMCs using anti-CD14 microbeads (Miltenyi-Biotec, Bergisch Gladbach, Germany). Purified monocytes were then suspended in medium consisting of 250 U/mL recombinant human (rh) IL-4 (R&D systems, Oxford, UK) and 50 ng/mL rhGM-CSF (R&D systems) in complete RPMI medium (10% FBS, 2 mM L-glutamine and 1% penicillin/streptomycin in RPMI). Cells were then cultured in 12-well plates for 6 days.

### Expression of cell surface markers

Expression of cell surface markers was assessed by addition of monoclonal antibodies, conjugated to the fluorophores FITC, PE or PC5, with specificity for cell surface markers. The selection of antibodies used depended on the markers of interest in each experiment and was chosen from the following: FITC-CD80 (clone MAB104, IgG1, Beckman Coulter), PC5-CD83 (clone HB15a, IgG2b, Beckman Coulter), PE-CD86 (clone HA5.2B7, IgG2bκ, Beckman Coulter), PE-CD206 (clone 3.29B1.10, IgG1, Beckman Coulter), PE-CD209 (clone AZND1, IgG1, Beckman Coulter), PC5-HLA-DR (Immu-357, IgG1, Beckman Coulter), FITC-CD54 (clone 84 H10, IgG1, Beckman Coulter) and PE-CD11c (clone BU15, IgG1, Beckman Coulter). Additionally, isotype controls were set up to control for non-specific binding. The following isotype controls were added, where relevant: FITC-IgG1 (Beckman Coulter), PE-IgG1 (Beckman Coulter), PC5-IgG1 (Beckman Coulter) and FITC-IgG2a (Beckman Coulter). One sample also remained unstained to provide an assessment of auto-fluorescence.

Cells were first washed with PBA (1% BSA (Sigma) and 0.02% sodium azide in PBS). 5 μL of each of the antibodies of interest was added to the cells and the resulting suspension mixed thoroughly before incubation for 30 min at 4°C in the dark. Cells were then washed with PBA, fixed by addition of 0.5% formaldehyde (Sigma) in PBS and then stored at 4°C until samples were analysed by flowcytometry.

### Collection and analysis of surface marker expression data

Data on cell surface marker expression were collected using a Beckman Coulter EPICS Altra™ (Beckman Coulter, High Wycombe, UK). Data were gated to include only cells of interest in the analysis, on the basis of forward and side scatter. The mean fluorescence intensity (MFI) value for each marker was recorded and the MFI value for the relevant isotype or unstained control was subtracted, to adjust for background fluorescence.

### Transglutaminase activity assay

Transglutaminase enzymatic activity was determined using a modified version of the hydroxamate assay previously described by Folk and Chung [16]. Briefly, a 25 μl portion of TG solution was mixed with 75 μl of Reagent A (0.2 M sodium acetate (pH 6.0), 0.03 M CBZ-Gln-Gly, 0.1 M hydroxylamine, 0.01 M glutathione, 5 mM CaCl2 and 5 mM DTT) in a well of a 96-well plate. Following incubation at 37°C for 10 min and to terminate the reaction, 75 μl of Reagent B (1 volume of 3 N HCl, 1 volume 12% trichloroacetic acid and 1 volume 5% FeCl3.6H2O (dissolved in 0.1 N HCl)) was added to the well. The resultant absorbance (492 nm) was measured using an Optima FLUOstar^® ^plate reader (BMG LabTech, Aylesbury, UK). A unit of transglutaminase activity is defined as the amount of enzyme catalysing the formation of 1 μmol of hydroxamic acid per minute under the described reaction conditions.

### Treating DCs with ECM proteins

In order to assess the effect of ECM protein treatment on DCs, cell culture plates were first coated with ECM proteins, followed by addition of DCs and assessment of markers of DC function at subsequent time points. 48-well plates (Corning Life Sciences) were coated with ECM proteins by addition of one of the following protein solutions: 0.01% FN (Sigma) in PBS, tissue transglutaminase 2 (TG-2; Sigma) at 0.039 U/mL (TG-2 [high]), 0.0036 U/mL (TG-2 [mid]) or 0.00022 U/mL (TG-2 [low]) in PBS to wells. Additionally, a PBS control and a BSA (1% in PBS; Sigma) control were set up. Plates were incubated at 37°C for 24 h. Each of the wells was then washed twice with cold PBS, before adding DCs in complete RPMI medium to each well. Cells were incubated at 37°C and assessed at 48 h and 72 h for cell viability and by flow cytometry (as described previously) for expression of the following surface markers: CD11c, CD80, CD86, CD83, HLA-DR, CD206, CD209 and CD54. Additionally, endocytic ability of cells after 48 h treatment was determined, as described below, and levels of IL-1α, IL-6, IL-8, IL-10 and IL-12 in culture supernatants were measured using the cytokine assay described below.

### Endocytosis

Assessment of DC endocytic ability was performed by measuring uptake of FITC-dextran (Invitrogen, Carlsbad, CA). DCs were first washed with PBS, followed by suspension in ice cold serum-free RPMI medium. The resulting suspension was then distributed evenly between four eppendorfs. FITC-dextran was added to two of the samples to a concentration of 1 mg/mL, while two remained untreated. One each of the FITC-dextran treated and untreated samples was then incubated in reduced light either at 37°C or at 4°C for 1.5 h. Following incubation, cells were washed with serum-free RPMI medium and subsequently with PBS. Finally, cells were fixed by addition of 0.5% formaldehyde in PBS and stored at 4°C until data collection was performed. Data were collected and analysed by flow cytometry as described previously. Data were gated on the basis of forward and side scatter to exclude dead cells.

### Scanning electron microscopy imaging (SEM)

Cells were treated with ECM proteins for 48 h as described above. Following treatment, cells were washed with PBS and then prepared for SEM imaging, as described elsewhere [17]. Briefly, samples were mounted onto aluminium SEM stubs using double-sided carbon tape and then gold coated, for 3 min, under an argon atmosphere before being imaged using a variable pressure JOEL JSM-6060LV SEM (Jeol, Tokyo, Japan) operating at an accelerating voltage of 10 kV. Image analysis was carried out using the in-built SEM Control User Interface software (version 6.57) and digital imaging system.

### Culturing ECM-treated DCs with T cells

DCs, pre-treated with different ECM proteins for 48 h as described previously, were cultured with autologous naïve T cells in order to establish the effect of ECM treatment on DC induction of a T cell response. Naïve T cells were isolated from CD14 depleted PBMCs, collected during the original positive selection of CD14+ PBMCs from which DCs were generated. In a two-step process, T cells were isolated from CD14 depleted PBMCs using a Pan T cell Kit (Miltenyi Biotec) and memory T cells were depleted on the basis of CD45RO expression, a memory T cell marker [18], using anti-CD45RO microbeads (Miltenyi Biotec). ECM treated DCs cells were incubated at 37°C for 45 min with gliadin (MP Biomedicals, Illkirch, France; 0.1% w/v in PBS with 0.1% v/v DMSO (Sigma)). Control DCs received no antigen. Following incubation, DCs were washed twice with PBS and autologous naïve T cells were added to DCs at a ratio of 5:1 (T cells to DC). DCs were also cultured without T cells where DCs received equal volume of T cell medium (RPMI enriched with 10% v/v serum from the original donor as well as 2 mM L-glutamine and 1% v/v penicillin/streptomycin), containing no T cell. Additionally, a culture of naïve T cells without DCs was established. Cells were incubated at 37°C for 5 days.

Supernatant was collected on day 5 and used to measure production of IFN-γ, IL-4, IL-10, IL- 12 and IL-13 using the cytokine assay described below. The cells were then washed with PBS and used in a T cell proliferation assay as described below.

### T cell proliferation

In order to determine T cell proliferation, incorporation of BrdU, an analogue of thymidine, into DNA in dividing cells was measured. Proliferation was measured using the BrdU Cell Proliferation Assay (Merck Chemicals, Nottingham, UK) carried out according to manufacturer's instructions.

### Cytokine assay

To assess the levels of cytokines released from cells, supernatant was collected and cytokines measured with a bead assay using FlowCytomix™ Basic and Simplex Kits (Bender MedSystems, Vienna, Austria). The assay was performed according to manufacturer's instructions and measured cytokines from a selection chosen from the following: IFN-γ, IL-4, IL-10, IL-12 and IL-13. Following assay preparation, data were collected using a Beckman Coulter FC500 and were analysed using FlowCytomix Pro 2.4 Software (Bender MedSystems).

### Statistical analysis

Means and standard deviations were calculated for each sample and differences between the means were compared using the Student *t*-test (two-tailed); p < 0.05 was considered significant.

## Results

### Pre-treatment with transglutaminase changed DC surface marker profile

**Figure 1:**
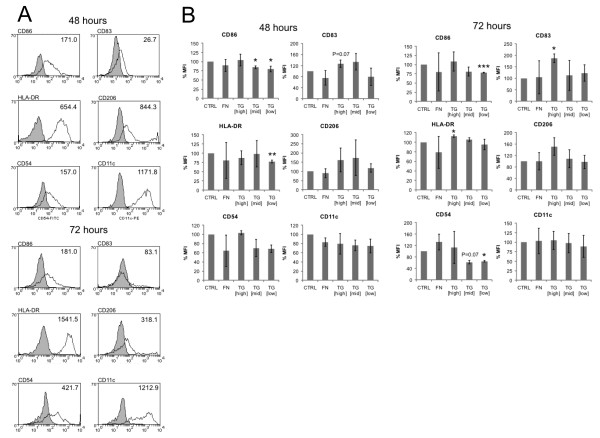
**Analysis of DC phenotype following treatment with ECM proteins**. **(A) **Flow cytometric analysis of the phenotype of untreated (control) DCs following culturing for 48 h (upper panel) and 72 h (lower panel). The filled histograms represent isotype or unstained controls, unfilled histograms show staining of named markers. The numbers are MFI values of a representative experiment **(B) **Phenotype of DCs untreated (CTRL) or treated with 0.01% FN, TG [high], TG [mid] or TG [low] for 48 h (upper panel) and 72 h (lower panel), (n = 3). MFI values for the indicated antigens are depicted as a percentage of the CTRL value. TG-2 activity defined as 0.039 U/mL (TG-2 [high]), 0.0036 U/mL (TG-2 [mid]) or 0.00022 U/mL (TG-2 [low]). Error bars represent standard deviations and * p < 0.05, ** p < 0.01, *** p < 0.001. NB: n = 2 for CD54 expression.

Enzymatically active TG-2, the activity of which was quantified by the hydroxamate assay (data not shown), was associated with dose-dependent changes in DC phenotype following 48 and 72 h of co-culture, as characterised by flow cytometry. Figure 1 demonstrates that culture of immature DCs with TG-2 [high] (0.039 U/mL TG-2) for 72 h led to a significant increase in some maturation markers (CD83, HLA-DR). Co-culture of DCs with TG-2 [mid] (0.0036 U/mL TG-2) was associated with significantly reduced CD86 expression at 48 h. Immature DCs which were exposed to TG-2 [low] (0.00022 U/mL TG-2) in culture had significantly reduced expression of CD86 and HLA-DR (P < 0.01) at 48 h and, additionally, a significantly lower expression of CD86 (P < 0.001) and CD54 at 72 h. Culturing in different conditions did not significantly affect cell viability [data not shown].

### Transglutaminase increased DC endocytic ability and altered cell morphology

**Figure 2:**
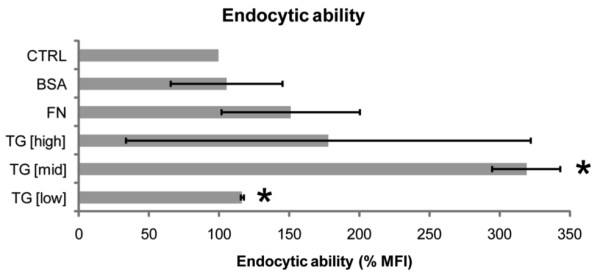
**Endocytic characteristics of DCs following treatment with ECM proteins**. Endocytic ability of the DCs, at 48 h, was assessed by the uptake of FITC-dextran using flow cytometry (n = 2). Data were gated, based on forward/side scatter characteristics and on FITC channel to exclude dead cells and contaminating clumps of FITC-dextran. Error bars represent standard deviations. * p < 0.05. NB: Statistical analysis is based on differences between means of untreated control (CTRL) and treated samples. Sample treatments correspond to untreated (CTRL), 0.01% FN, 1% BSA and TG-2 activity defined as 0.039 U/mL (TG-2 [high]), 0.0036 U/mL (TG-2 [mid]) or 0.00022 U/mL (TG-2 [low]).

**Figure 3:**
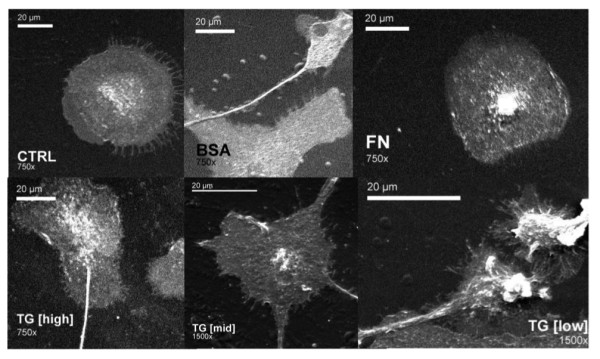
**Scanning electron microscopy (SEM) images of DCs following treatment with ECM proteins**. Dendritic cells, following 48 h treatment with TG[high], TG [mid] or TG [low] were mounted onto aluminium stubs, gold coated under an argon atmosphere before being imaged using a JOEL 6060LV variable pressure SEM operating at 10 kV accelerating voltage at x750 and x1500 magnifications (n = 1). Sample treatments correspond to untreated (CTRL) and TG-2 activity defined as 0.039 U/mL (TG-2 [high]), 0.0036 U/mL (TG-2 [mid]) or 0.00022 U/mL (TG-2 [low]).

Endocytic ability is key to the function of immature DCs, allowing capture of antigens for later presentation to T cells. Uptake of FITC-dextran by DCs cultured with different concentrations of TG-2 was used to quantify endocytic ability. Figure 2 demonstrates that treatment with TG-2 [mid] or TG-2 [low] for 48 h significantly (P < 0.05) increased the endocytic ability of DCs. TG-2 [high] showed large variability in its effect, showing a wide standard deviation, implying little effect on the endocytic ability of DCs. TG-2 also affected the morphology of DCs, in a concentration-dependent manner, as shown in Figure 3. TG-2 [high] was associated with a reduced number of dendrites but which were longer in comparison with TG-2 [mid] and TG-2 [low]. TG-2 [mid]-treated DCs had long, broad dendrites, while TG-2 [low] lead to more numerous, but shorter and thinner dendrites.

### Transglutaminase had a concentration-dependent effect on DC induction of T cell proliferation and cytokine profiles

**Figure 4:**
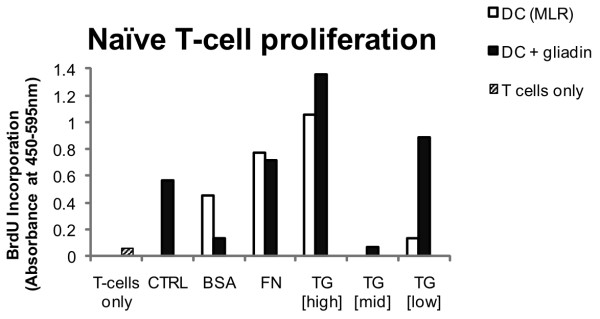
**T-cell proliferation in response to co-culture with treated DCs**. Naïve T-cell proliferation was assessed following 5 days of culture with pre-treated DCs (48 h and loaded with gliadin) (n = 1). Samples include naïve T-cell cultured in a mixed lymphocyte reaction (MLR) and treated DCs, and naïve T cells in single culture. Absorbance values for DCs in single culture were subtracted to give the values shown. Sample treatments correspond to untreated (CTRL) and TG-2 activity defined as 0.039 U/mL (TG-2 [high]), 0.0036 U/mL (TG-2 [mid]) or 0.00022 U/mL (TG-2 [low]).

**Figure 5:**
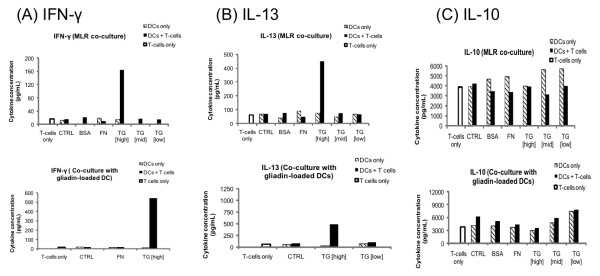
**Cytokine expression profile of naïve T cells in co-culture with dendritic cells**. Cytokine production from co-culture of naïve T cells and DCs following treatment with TG as previously described (n = 1). Concentrations (pg/ml) of IFN-γ (A), IL-13 (B) and IL-10 (C) in supernatant following co-culture of naïve T cells with DCs in a mixed lymphocyte reaction (MLR) (upper panel) or co-culture of naïve T cells with DCs which had been loaded with gliadin. Also depicted is cytokine production by naïve Tcells and DCs in single culture. Treatments correspond to untreated (CTRL), 1% BSA and TG-2 activity defined as 0.039 U/mL (TG-2 [high]), 0.0036 U/mL (TG-2 [mid]) or 0.00022 U/mL (TG-2 [low]).

To establish whether observed changes in phenotype and function might have an effect on DC induction of T cell responses in either mixed-lymphocyte reaction (MLR) or antigen-specific (DCs loaded with gliadin, the major gluten antigen) systems, TG-2- treated DC were cultured with autologous naïve T cells and T cell proliferation (Figure 4) and production of IFN-γ, IL-13 and IL-10 were measured (Figure 5). TG-2 showed a concentration-dependent effect on the induction of naïve T cell proliferation by DCs. The data indicate that, as expected, naïve T cells were unable to proliferate in the absence of DCs, as shown by the low BrdU absorbance for T cell only cultures. Naïve T cells co-cultured with TG-2 [high]-treated DCs in a MLR experiment showed increased proliferation compared to those co-cultured with untreated (control) DCs. These T cells also appeared to produce larger amounts of IFN-γ and IL-13, while IL-10 production was reduced. TG-2 [mid]-treated DCs showed reduced induction of naïve T cell in an MLR experiment, while TG-2 [low]-treated DCs appeared to be slightly enhanced in induction of naïve T cells. TG-2 [mid]- and TG-2 [low]-treated DCs seemed to reduce IL-10 production by naïve T cells in co-culture. Interestingly, treatment with both concentrations was associated with a small increase in production of IL-10 by DCs, however. Neither TG-2 [mid] nor TG-2 [low] treatments seemed to have an effect on DC induction of IFN-γ or IL-13 production by naïve T cells in MLR experiments.

Proliferation of naïve T cells appeared to be further enhanced when these cells were cocultured with gliadin-loaded TG-2 [high]-treated DCs. Co-culturing of naïve T cells with TG-2 [low]-treated DCs loaded with gliadin also increased T cell proliferation, while T cell proliferation was reduced in co-culture with TG-2 [mid]-treated gliadin loaded DCs. When naïve T cells were cultured with TG-2 [high]-treated DCs which had been loaded with gliadin, IFN-γ and IL-13 production by T cells were raised considerably. At the same time, IL-10 production by naïve T cells was reduced. IFN-γ and IL-13 production by naïve T cells were unaffected when these cells were in co-culture with TG-2 [mid]- or TG-2 [low]-treated DCs, loaded with gliadin. Naïve T cell production of IL-10 was reduced in co-culture with gliadin-loaded DCs previously treated with TG-2 [mid] or TG-2 [low]. However, under similar conditions, TG-2 [low]-treated DCs appeared to produce substantially more IL-10 than control DCs. IL-12 was not detected in any samples, while levels of IL-4 were negligible and production by naïve T cells was unaffected by co-culture with TG2treated DCs (data not shown).

## Discussion

The observations in this study (summarised in Table 1) demonstrate that DCs cultured with different concentrations of TG-2 showed changes in their phenotype, function and induction of T cell activation at different timepoints. TG-2 used in this study was a commercial preparation derived from guinea pig liver and demonstrated a high degree of enzymatic activity. It has previously been reported that human TG-2 and TG-2 from guinea pig liverare highly comparable in detection of human anti-TG2 autoantibodies when used in a diagnostic test for coeliac disease [19]. Therefore, it was reasonable to assume that the two proteins are similar enough for the effects of guinea pig TG-2 on DCs to be comparable to those of human TG-2. However, given that more recently human TG-2 has become more readily available future work in this area would clearly benefit from the use of human TG-2 in such experiments. Moreover, how the activity of the commercial preparation of TG-2 corresponds to the activity of TG-2 in the human small intestine is poorly established; indeed little is known about the activity and levels of TG-2 in the small intestine. Thus, a range of TG-2 concentrations was used to screen for the effect of TG-2 on DCs. Establishing the range of TG-2 concentration in the small intestine in normal and coeliac patients would therefore be extremely beneficial for studies in this area.

**Table 1:** A summary of the effects of ECM protein treatment on DC phenotype, morphology and function

	FN	TG [high]	TG [mid]	TG [low]
***Surface marker profile***				

Antigen-presentation (HLA-DR)	-	↑*^72^	-	↓*^48^

Co-stimulation (CD86)	-	-	↓*^48^	↓*

Endocytosis (CD206)	-	-	-	-

Maturity (CD83)	-	↑*^72^	-	-

Adhesion (CD54)	↑	-	↓	↓*^72^

***Morphology***	-	Mature	-	Immature

***Function***				

Endocytic ability	↑	-	↑*	↑*

T-cell stimulation	-	↑	↓	↓

T-cell polarisation	-	Effector	Tolerance	-

* indicates that change was significantly different from the control. For phenotypic results, *48 indicates significance only at 48 h, *72 indicates significance only at 72 h

The data in this study show that DCs cultured with TG-2 gained a different phenotype compared to control DCs, and also compared to DCs treated with FN, a structural ECM protein. The data indicate that TG-2 acted in a concentration-dependent manner, with different surface marker profiles associated with each of the concentrations of TG-2. The function of immature DCs is to capture antigens and, once activated by molecular patterns associated with pathogens, to migrate to lymphoid tissue where they activate T cells [2]. Thus, immature and mature DCs can be contrasted on the basis of their surface phenotype and certain functional properties [20]. While immature DCs are associated with high levels of endocytic receptors, such as CD206 and CD209, and thus show enhanced endocytic ability [20], mature DCs have high expression of molecules required to interact with and stimulate T cells - MHC class II (HLA-DR), co-stimulatory molecules (CD80, CD86), the adhesion molecule, CD54 (2) and the maturity marker, CD83 [20,21]. The data demonstrate that DCs cultured with high concentration TG-2 gained a more mature phenotype, as shown by increased expression of maturation markers (CD83, MHC class II) after 72 h culturing. The data may indicate initiation of this maturation process at 48 h by high concentration TG-2, where there was an almost significant increase in expression of the maturation marker CD83, although other markers were unaffected. However, the presence of lower concentrations of TG- 2 was associated with the converse effect. DCs appeared to acquire a more immature phenotype when cultured with lower concentrations of TG-2 (TG-2 [mid]/TG-2 [low]), which is demonstrated by a reduction in the expression of markers of maturation at 48 h (CD86, MHC II) and 72 h (CD83, CD54) and also by enhancement of endocytic ability of DCs.

Furthurmore, our data indicate that the presence of TG-2 affected DC morphology. DCs cultured with high concentration TG-2 had a reduced number of dendrites, but which were longer and thinner in comparison to lower concentrations of TG-2. The presence of lower concentrations of TG-2 resulted in DCs having a considerable number of very short dendrites. Dendrites are important in several aspects of DC function. Immature DCs have less pronounced dendrites and these extrusions of the cell surface are thought to contribute to increasing surface area as a means of augmenting the efficiency of antigen uptake [22]. On the other hand, the long dendrites associated with mature DCs [22] may be involved in allowing DCs to form interactions with a multitude of T cells with distinct specificities within lymphoid tissue, in order to initiate adaptive immune responses with high efficiency. Thus, DCs treated with high concentration may have taken on a more mature morphology, while treatment with lower concentrations may have led to a more immature morphology, which clearly supports the data collected on surface phenotype.

The role of DCs *in vivo *is to stimulate adaptive immune responses, by presenting antigens to T cells [2]. DCs treated with high concentration TG-2 were ameliorated in their ability to trigger proliferation of naïve T cells in an MLR experiment. Their ability was further increased by antigen loading in the form of gliadin (the major gluten antigen). DCs treated with high concentration TG-2 showed a more mature surface phenotype, which could account for the observed increases in T cell proliferation. Cytokine production data from the same experiment supported these observations. The data indicated that DCs pre-treated with high concentration TG-2 led to high-level cytokine production by T cells, characteristic of an effector T cell response, both in MLR experiments and when these DCs had been loaded with gliadin. The balance of T cell cytokines is important in determining the nature of an effector T cell response, since T cell subsets display reciprocal regulation [23]; initiation of a potent Th1 response, leading to high-level IFN-γ production prevents Th2 expansion [18]. Similarly, rapid early proliferation of Th2 cells, leading to cytokines such as IL-4 and IL-13 [4], inhibits Th1 development and both Th1 and Th2 subsets are thought to inhibit Th17 expansion [18]. However, polarisation of a specific T cell response towards a particular subset is only thought to become dominant in long-standing infections [18] and therefore early naïve T cell responses are likely show more mixed cytokine profiles, which were evident in this study. Moreover, it has previously been reported that in healthy subjects, stimulation of naïve T cells with gliadin results in generation of a Th0 type response, represented by a mixed cytokine profile [15].

Treatment of DCs with lower concentrations of TG-2 was associated with a decrease in naïve T cell proliferation, both in MLR experiments and when the TG-2-treated DCs had been loaded with gliadin. Interaction of naïve T cells with immature DCs, where antigens are presented without co-stimulation, is associated with induction of anergy or tolerance [6,18]. Culturing DCs with low concentrations of TG-2 led to a more immature DC phenotype. These data may indicate, therefore, that the DCs which had been pre-treated with low concentration TG-2 induced anergy in T cells with which they interacted. Treatment with lower concentrations of TG-2 (TG-2 [mid]/TG-2 [low]) also resulted in increased DC production of IL-10, although T cell production of IL-10 was slightly reduced. IL-10 is an anti-inflammatory cytokine associated with regulatory T cells (Treg) [18], which serve to limit the inflammation caused by effector T cell-mediated responses, in particular those mediated by Th1 cells [6,18]. It has also been found that IL-10 is required for promoting tolerance in the intestinal immune system, mediated by Treg cells [24]. Interestingly, IL-10 production by T cells was considerably reduced when they were cultured with TG-2 [high]-treated DCs. Taken together, these data could indicate that DCs exposed to low concentration TG-2 were tolerogenic, whereas exposure to high concentration TG-2 breaks this tolerance, leading to induction of T cell responses. Although these results represent data from only one experiment, they do support other findings from this study and provide an indication of the effect of TG-2 pre-treatment on DC stimulation and polarisation of naïve T cells. In future work, it would be worthwhile to ascertain the effect of TG-2 treatment on DC ability to induce a Th17 response, by measuring IL-17 levels in a similar co-culture experiment, particularly given the emerging importance of Th17 cells in autoimmune disease [25]. Additionally, a co-culture experiment over a longer time course would be beneficial.

TG-2 is an enzyme involved in cross-linking proteins containing glutamine residues, with a physiological role in ECM stabilisation, in part by giving rise to resistance to degradation [26]. Interestingly, a recent study found that an intact ECM is associated with promoting T cell tolerance [27]. Although this study looked at the effect of an intact ECM directly on T cells, it would be reasonable to speculate that the stability of the ECM, to which TG-2 contributes, could also have a tolerogenic effect on DCs. Several possible mechanisms for the extra-cellular effects of TG-2 on DCs found in this study are also plausible. Previous work has looked at the interaction between TG-2 and integrins, particularly as a mediator between these molecules and fibronectin [28]. It has also been reported that the integrins (β_1 _and β_3_), with which TG-2 is able to interact, are expressed by DCs [2,29,30]. In this way they may affect cell adhesion and migration - one previous study reported that β_1 _integrins can affect DC morphology [31]. Interactions with the extra-cellular matrix may also be important. In recent work, the effect of DC adhesions on phenotype was studied [30]. This study found that the increased strength of DC adhesion to the ECM resulted in greater pro-inflammatory properties, with an increase in the expression of maturation markers and release of pro-inflammatory cytokines. TG-2 also interacts with surface G-protein-coupled receptors to modulate intracellular protein kinase pathways, which have the potential to alter DC intracellular processes [28,32]. TG-2 is also able to convert TGF-β from an inactive to an active form [33], which might contribute to TG-2's effects on the ECM and perhaps also immune responses. Deciphering how these processes could interact to explain the results in this study is complex. It is conceivable that at least two systems are at work, with differing affinities for TG-2, which could explain the dose-dependent effects noted. For example, a high-affinity system leading to greater endocytosis, keeping DCs in a surveillance role and a low-affinity system leading to maturation and migration, which is able to dominate the high-affinity system. Studying these mechanisms would be an extremely informative area of investigation in future work.

Dysregulation of TG-2 is implicated in the pathogenesis of several diseases such as coeliac disease and neurodegeneration [9]. Although the enzymatic activity of TG-2 and, to some extent, its physiological roles in ECM stabilisation, apoptosis and interactions between cells and the ECM are well-established, less is known about its interactions with pathological processes [9]. TG-2 has previously been identified as an autoantigen in coeliac disease [11] and antibodies directed against it can be detected as the basis of a diagnostic test for coeliac disease [34]. TG-2 is found in the small intestinal submucosa of both healthy subjects and also those with coeliac disease, and levels of it are elevated in the small intestine of those with coeliac disease [12]. The abdominal symptoms and malabsorption found in coeliac disease are caused by inflammation of the small intestine which results in loss of villi [35]. The inflammation associated with coeliac disease is mediated by IFN-γ [18], released by Th1 cells specific for gluten-derived antigens [13]. These T cells in turn are stimulated by DCs, the function of which is to serve as a link between the innate and adaptive immune systems. Thus, processes which alter interactions between DCs and T cells are of potential interest in determining the pathogenesis of conditions such as coeliac disease.

In the context of coeliac disease, data in this study may support a model in which low concentrations of TG-2, in the healthy small intestine, promote tolerance to gut antigens, such as gluten, therefore preventing T cell mediated responses associated with autoimmune disease [36]. Previous work has found that levels of TG-2 are raised in the small intestine of coeliac disease patients [12]; it is possible that this is associated with damage to the gut epithelium causing release of TG-2 either from sequestration or as a cellular stress response [12]. The resulting high concentrations of TG-2 may then alter DC phenotype, as observed in this study, leading to an enhanced ability to trigger T cell responses to gluten antigens. Autoantibodies to TG-2 generated during the adaptive response [11] may then cause further damage and release of TG-2 leading to a vicious circle of inflammation. Aspects of this model might be applicable in other immunologically-mediated conditions in which DCs have a role or may have a more general role in the initiation of an immune response. Clearly, it will be necessary to do further work to clarify the effect of TG-2 on DCs, in particular to examine the mechanisms by which TG-2 interacts with, and alters, DC phenotype and function. It would also be of interest to repeat experiments carried out in this study using DCs from coeliac disease patients in order to develop a more comprehensive model of the interactions between TG-2 and DCs involved in the pathogenesis of coeliac disease in vivo. Modelling of these interactions could be of potential benefit in developing a better understanding of the processes which give rise to coeliac disease and also of therapeutic benefit in treating coeliac disease, a condition for which currently the only therapy is to exclude gluten from the diet [13].

## Conclusions

This study indicates that tissue transglutaminase has a concentration-dependent effect on DCs; higher concentrations were associated with a more mature phenotype and increased ability to stimulate T cells, while lower concentrations led to maintenance of an immature phenotype. While further work will be required to elucidate the molecular basis of observed transglutaminase induced changes in DCs, these data provide support for an additional role for transglutaminase in coeliac disease and demonstrate the potential of *in vitro *modelling of certain aspects of coeliac disease pathogenesis.

## Competing interests

The authors have declared that no competing interests exist.

## Authors' contributions

WJD design of experiments, cell culture, DC work, T-cell work, flow cytometry, drafting of manuscript; DYSC design of experiments, cell culture, TG activity, DC work, flow cytometry; AMG: design of experiments, funding. All authors read and approved the final manuscript.
